# NAMPT as a Dedifferentiation-Inducer Gene: NAD^+^ as Core Axis for Glioma Cancer Stem-Like Cells Maintenance

**DOI:** 10.3389/fonc.2019.00292

**Published:** 2019-05-02

**Authors:** Antonio Lucena-Cacace, Masayuki Umeda, Lola E. Navas, Amancio Carnero

**Affiliations:** ^1^Department of Cell Growth and Differentiation, Center for iPS Cell Research and Application, Kyoto University, Kyoto, Japan; ^2^CIBERONC, ISCIII, Madrid, Spain; ^3^Instituto de Biomedicina de Sevilla (IBIS), Hospital Universitario Virgen del Rocío (HUVR), CSIC, Universidad de Sevilla, Sevilla, Spain

**Keywords:** NAD, NAMPT, Glioma, GSCs, PARP, SIRT, TMZ, GBM

## Abstract

Glioma Cancer Stem-Like Cells (GSCs) are a small subset of CD133^+^ cells with self-renewal properties and capable of initiating new tumors contributing to Glioma progression, maintenance, hierarchy, and complexity. GSCs are highly resistant to chemo and radiotherapy. These cells are believed to be responsible for tumor relapses and patients' fatal outcome after developing a recurrent Glioblastoma (GBM) or High Grade Glioma (HGG). GSCs are cells under replicative stress with high demands on NAD^+^ supply to repair DNA, maintain self-renewal capacity and to induce tumor plasticity. NAD^+^ feeds Poly-ADP polymerases (PARP) and NAD^+^-dependent deacetylases (SIRTUINS) contributing to GSC phenotype. This energetic core axis is mainly controlled by the rate-limiting enzyme nicotinamide phosphoribosyltransferase (NAMPT), an important oncogene contributing to tumor dedifferentiation. Targeting GSCs depicts a new frontier in Glioma therapy; hence NAMPT could represent a key regulator for GSCs maintenance. Its inhibition may attenuate GSCs properties by decreasing NAD^+^ supply, consequently contributing to a better outcome together with current therapies for Glioma control.

## Introduction

Gliomas are the most prevalent primary brain cancer in adults. Arising from glia cells, Gliomas involve a broad category of tumors including astrocytoma, oligodendroglioma, and glioblastoma (GBM). Regardless of tumor aggressiveness, malignancy and infiltration, these glia-derived tumors rarely exceed a median survival time of 12–14 months (1, 2). Driven by the infiltrative nature of these tumors, the clinical approach is difficult and relapses often occur with fatal consequences.

Therapeutic responses and patient survival rely on intratumoral heterogeneity ruled by genetic and epigenetic effectors. Nonetheless, there are many physiological barriers to the development of successful treatments. Blood-brain barrier (BBB) is a major limitation when it comes to deliver a chemotherapy-based treatment. Surgical resection is an ineffective long-term procedure since Gliomas infiltrate together with healthy brain tissue and their resection become virtually impossible. Invasive procedures compromise the patient's life quality and radiotherapy drives harmful side effects. As a final outcome, gliomas ultimately relapse in highly radio- and chemo-resistant forms.

These unsuccessful attempts to control glioma's fate have fostered research looking for more effective therapies.

Glioma Stem-like cells (GSCs) are Cancer initiating cells (CICs) maintaining self-renewal properties, sustaining cellular hierarchy, and partially explaining the tumor heterogeneity since they maintain a progeny responsible for tumor complexity (3, 4). GSCs express certain stem cell-like markers and were first characterized as CD133^+^ cells able to initiate new tumors in mice (5). GSCs are chemo- and radio resistant, contributing to tumor relapse. Hence, targeting the molecular networks controlling GSCs maintenance is a promising new treatment frontier for glioma control.

**Figure 1:**
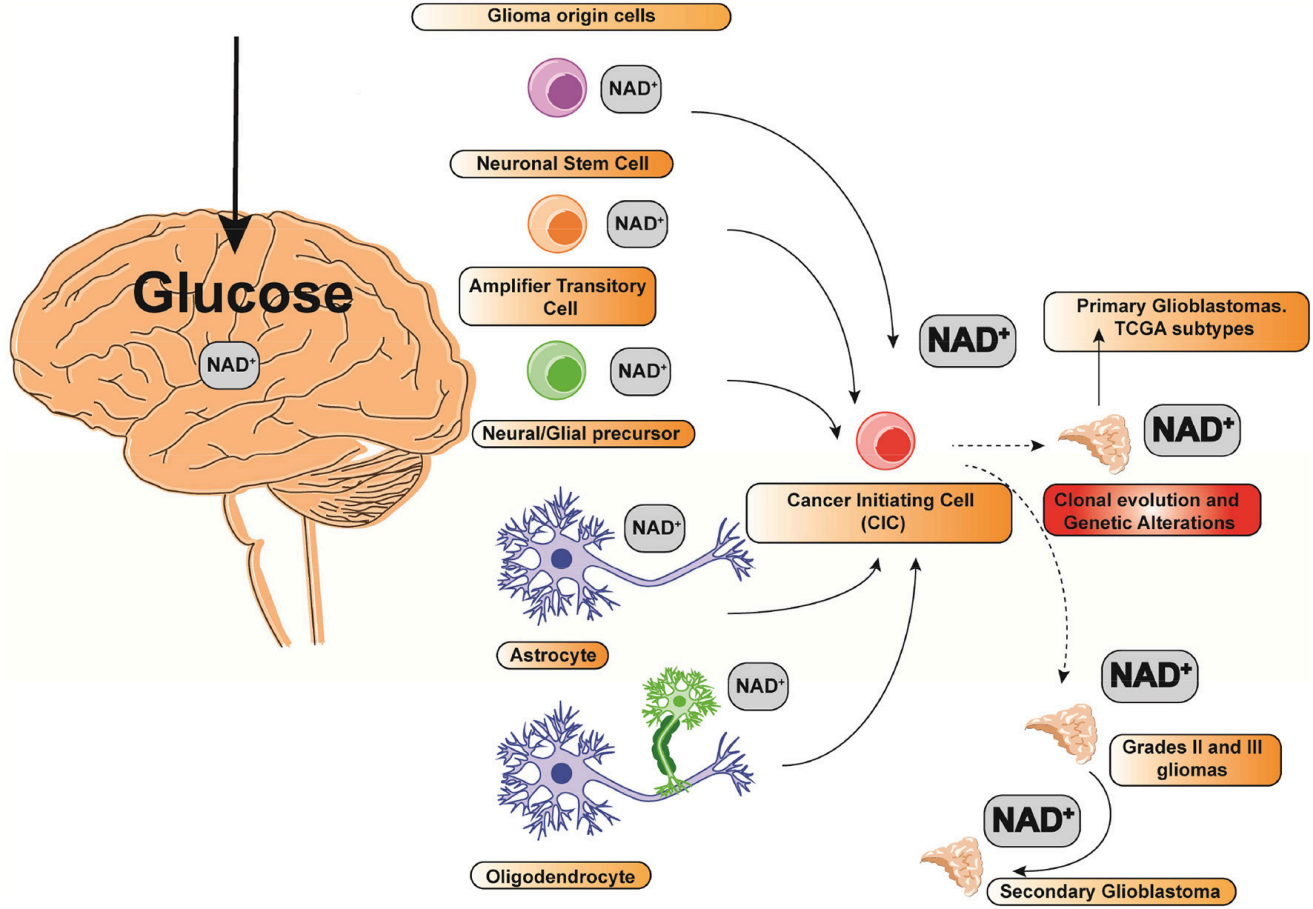
Glioma cell(s) of origin and NAD^+^ relative consumption. The size of the font indicates the relative NAD^+^ consumption.

According to the most recent World Health Organization for Glioma classification, GBM is labeled as a grade IV glioma (6). Although GBM often originates from 5 different cell types, neuronal stem cells, transit amplification cells, glial/neural progenitors, astrocytes, and oligodendrocytes (Figure 1), it may be classified into two types depending on its severity: primary and secondary GBM. A low grade diffuse astrocytoma (Grade II glioma) or an anaplastic astrocytoma (Grade III glioma) might evolve to a secondary GBM (Figure 1). Counting up to 95% of total cases, primary GBM tumors are the most frequent. Since primary and secondary GBMs display a markedly micro vascularization and high mitotic activity, they are mostly indistinguishable histologically. High mitotic activity turns Gliomas in very fast growing tumors. Since the growth rate is often greater than its angiogenesis capacity, the tumor's core is often hypoxic and necrotic (7). Nonetheless, secondary GBMs only count for 5% of the total cases.

Representing up to 16% of brain tumors, GBM risk and incidence are age-related. Normally it affects an average of 7.2 individuals per 100,000 adults aged 19 and above. Nonetheless, 14.6 individuals per 100,000 are aged between 74 and 84, representing the highest incidence peak (7).

Regardless of gender, GBM patient prognosis rarely exceeds 12 months after diagnosis. Nonetheless, GBM shows gender preference, being 40% more frequent in men (1, 2). Broadly, the standard therapeutic strategy for Gliomas consists of a primary resection surgery followed by radiotherapy and chemotherapy. However, since surgery is virtually unable to remove the neoplastic tissue, GSCs will ultimately reconstitute the whole tumor, leading to the consequence that 100% of patients end up with a relapse of the tumor in a peripheral area up to 2 cm from the focus of origin.

Following surgery, radiotherapy is often applied with Temozolomide (TMZ) as an adjuvant treatment. TMZ is a DNA alkylating agent able to permeate through the brain blood barrier (8). Since the early 2000s, it has been one of the few FDA approved drugs for GBM control, providing a survival advantage of 2.5 months. Recently, FDA approved Bevacizumab and Lomustine for recurrent GBM and Carmustine wafer for newly diagnosed high grade-malignant glioma (HGG) and recurrent GBM. To date, all these new treatments show inconclusive results regarding the benefits or survival advantages over TMZ, merely providing disease-related symptoms.

Even though treatments in other cancers have greatly evolved in recent years, the clinical approach to Glioma treatment remains challenging. Despite treatment, < 5% of patients survive more than 5 years after the diagnosis.

An unmonitored cluster of gene expression data from 200 GBM samples from TCGA in 2011 established four subtypes of GBM according to their molecular profile: Proneural, Classical, Mesenchymal, and Neural (9, 10).

Proneural subtype is characterized by abnormalities in platelet-derived growth factor alpha receptor (PDGFRA) or in isocitrate dehydrogenase 1 (IDH1). The Classical subtype is mainly characterized by mutations in the epidermal growth factor receptor (EGFR). Mesenchymal subtype features mutations in neurofibromine 1 (NF1). Neural subtype was not completely defined but it is known to contain amplification and overexpression of EGFR. Nonetheless, molecular markers defining the neural subtype of GBM could be contaminated with normal neural tissue in the tumor margin, thus not representing a true subtype.

The mutational spectrum of GBM is varied. They are highly molecularly complex tumors and the number of coding mutations per tumor sample is assorted.

Ranging from 3 to 179 mutations per tumor, the average mutational burden per tumor is 53 (9). The most frequent mutations in GBMs are PTEN (29%), TP53 (29%), EGFR (20%), 21 NF1 (9%), RB1 (8%), phosphatidylinositol-4,5-bisphosphate alpha catalytic subunit - 3- kinase (PIK3CA; 7%), regulatory subunit 1 of 3-phosphoinositide (PIK3R1; 6%), and IDH1(5%) (11, 12).

All GBM subtypes and HGGs have in common a deep dependence on glucose consumption as the main source of metabolic energy. Gliomas are highly metabolic and rely on glycolytic pathways. This is why most gliomas may have a strong dependence on NAD^+^ metabolism as the main intermediary in the reduction-oxidation reactions (13, 14). Additionally, GSCs are cells under replicative stress with high demands on NAD^+^ supply to repair DNA, maintain self-renewal capacity and to induce tumor plasticity. NAD^+^ feeds PARP and SIRTUINS contributing to GSC phenotype. This energetic core axis is mainly controlled by the rate-limiting enzyme NAMPT, an important oncogene contributing to tumor dedifferentiation.

In addition, NAMPT is particularly overexpressed in mesenchymal GSCs, where its expression is correlated to a hypomethylation state driven by depletion of methionine and *de novo* methyltransferases, sustaining mesenchymal GSCs rapid growth and NAM consumption to support NAD^+^ utilization and sustain DNA hypomethylation (15), another marker of poor GBM prognosis.

In this review we will explore the importance of NAD^+^ as core axis for Glioma Cancer Stem-Like Cells maintenance and how targeting NAMPT over GSCs could represent a promising new frontier therapy for Glioma control.

## Malignant Clonal Evolution on Gliomas

Intertumoral diversity relies on a particular clonal evolution driven by a cell of origin acquiring cancer-initiating capacity (Figure 1) (16). Whether specific glioma cells of origin are susceptible to certain cancer-initiating mutations driving to GSCs is unclear. Nonetheless, there are many studies suggesting that certain progenitors and stem cells are markedly susceptible to a variety of different mutations (Figure 1) (17). Some cells of origins might potentially display a preferential vulnerability to specific mutations. To note, TCGA molecular subtypes are augmented for lineage markers characteristic of distinct glia-derived cell types, suggesting that molecular and/or epigenetic profiles of the Glioma initiating cell are maintained during tumorigenesis (18). Clonal evolution on Gliomas can be an important determinant defining tumor phenotype and genotype in both GBM and HGGs. Nonetheless, given the diversity of driver mutations representing different subtypes of GBM, the contribution of the cancer initiating cell is unknown (Figure 1) (11). Indeed, recent studies in adult neural stem cells (NSCs) and oligodendrocyte precursor cells (OPCs) reveal that they might have different transformation capacity as a cellular origin candidacy since their self-renewal capacity in the adult human brain is again under re-evaluation (19). Any cell type out of the five candidates to drive into a primary GBM can evolve to a defined molecular GBM subtype, mainly depending on their driver mutation leading to the cancer-initiating phenotype (19) (Figure 1). Since single GBM cells are not mere genetic phenocopies, analyses from tumor cells taken from the same patient show notably heterogeneous tumors consisting of mutant cells carrying different genetic burden, expression of different cell markers and different levels of aneuploidy (20). The inherent heterogeneity and complexity of GBM, HGG, and GSCs show a variable expression of transcriptional programs embracing different cellular processes involving cell cycle, hypoxia, and immune signaling (10).

Alterations such as chromosomal aberrations, genomic rearrangements, and focal copy number aberrations can give rise to GSCs and eventually to a glioma.

GSCs are a subpopulation of cells that explain part of tumor heterogeneity. Circulating GSCs also display stem cell-like properties (21). They are cells with capacity for differentiation and self-renewal, responsible for the hierarchical clonal development and able to regenerate *de novo* tumor from a single cell. The metabolism of NAD^+^ could play a relevant role in the mechanisms associated with chemoresistance in CICs and their maintenance mechanisms.

## Nicotinamide Adenine Dinucleotide Metabolism

Four major molecules are used as substrates for the synthesis of NAD^+^. These molecules are dietary tryptophan (L-Trp), nicotinic acid (NA), nicotinamide (NAM), and nicotinamide riboside (NR) (22).

**Figure 2:**
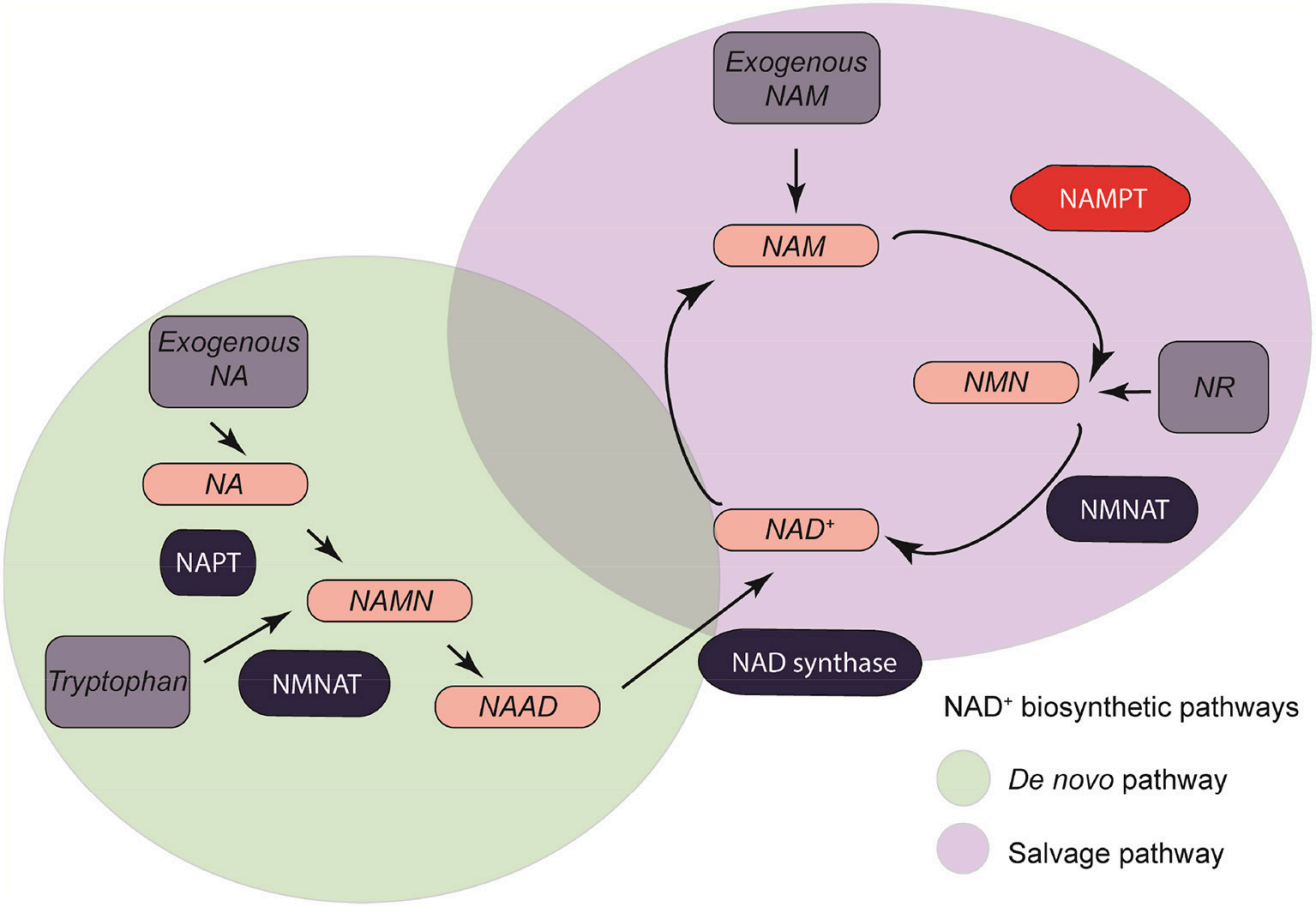
NAMPT/Nicotinamide adenine dinucleotide metabolism. Four major synthesis precursors (Exogenous NA, exogenous NAM, NR, and Tryptophan, dark gray) are divided between two major pathways: *de novo* pathway (light green), and salvage pathway (light purple).

These four major metabolic molecules are involved in the synthesis of NAD^+^ through two major pathways: *De Novo* Pathway and Salvage Pathway. Some metabolic intermediates such as nicotinamide mononucleotide (NMN) might also stimulate the direct synthesis of NAD^+^ (Figure 2) (23, 24).

*De novo* synthesis of NAD^+^ takes place intracellularly in an eight-step reaction (25–29). This pathway takes the L-Trp acquired through daily diet as a conversion molecule when obtaining NAD^+^. Tryptophan-derived quinolinic acid is produced and used by quinolinate phosphoribosyl transferase (NAPT) to form nicotinic acid mononucleotide (NAMN). NAMN is converted to nicotinic acid adenine mononucleotide (NAAD) in a NMN adenylyltransferase (NMNAT) -mediated reaction with ATP consumption (25). There are three isoforms of NMNAT (NMNAT 1-3) with different tissue and cellular locations depending on the metabolic requirements (30). NMNAT-1 is an ubiquitously expressed nuclear protein. NMNAT-2 is normally present in the golgi apparatus and the cytosol (31–35). NMNAT-3 may be present in both the cytosol and mitochondrial compartments. The efficiency of obtaining NAD^+^ by dietary tryptophan is very low compared to that obtained by the Salvage pathway.

This NAD^+^ synthesis pathway is also known as the Preiss-Handler pathway. In this pathway the NAD^+^ is generated from Niacin (Vitamin B3) in a three-step reaction (36). NAM can also be a precursor of NAD^+^ through its conversion to NMN by the limiting enzyme nicotinamide phosphoribosyl transferase (NAMPT). NMN acts as an intermediate by catalyzing the reversible addition of a ribose group from 5-phosphoribosyl-D-ribosyl-1-pyrophosphate to NAM. In the mitochondrial respiratory chain, NADH acts as the main donor of electrons, which end up in the generation of ATP by oxidative phosphorylation (37–39).

The liver is the major organ involving NAD^+^ metabolic activity as it expresses all enzymes for either metabolism or recycling. Hepatic cells can actually convert all its precursors: NA, NAM, and their ribosides as well as L-Trp. For that, NAMPT and NAPRT mRNA levels are particularly high in the liver, so this organ is one of the major NAD^+^ recycling and synthesis engine cores in humans (40, 41).

All tissues have the potential to at least convert NAM and NR into NAD^+^. That is why the NAMPT enzyme and at least two NMNAT isoforms are ubiquitously expressed in all cells and tissues. Some others, such as NMNAT2, are brain specific (42). Other NMNAT isoforms are expressed in a greater proportion in the pancreas, thyroid gland and lymphocytes (31). In mammalian cells, the metabolism of NAD^+^ is compartmentalized. The formation of NAD^+^ from NMN takes place in the nucleus and in the mitochondria (37, 38, 43). These two organelles are particularly important as the most important NAD^+^-dependent intracellular signaling pathways occur in them.

The most important cytosolic NAD^+^ precursor for mitochondrial synthesis is NMN. Therefore, NAD^+^-dependent cellular processes are intimately linked to the most important molecular events that could lead to cancer: genomic alterations, metabolic imbalances, and changes in the transcription patterns of candidate oncogenes or tumor suppressors.

Nicotinamide phosphoribosyl transferase (NAMPT) is the limiting enzyme of the NAD^+^ Salvage Pathway, the major NAD^+^ source in living cells, being the major contributor to NAD^+^ maintenance, recycling, and homeostasis.

NAMPT is a highly conserved protein among mammals. It was cloned and isolated for the first time in the organism Haemophilus ducreyi and it has been extensively studied.

It was originally characterized as the human homologous protein pre-B cell colony-enhancer factor (PBEF). In its role as PBEF it acts as a cytokine, which stimulates early B cell formation in a synergistic effect with interleukin 7 (IL-7) and stem cell factor (SFC) (44, 45).

The gene encoding NAMPT is found on human chromosome 7, specifically at the 7q22.3 locus. The size of the gene within the DNA is 3.7 kilobases (kb) and contains 11 exons and 10 introns that encode a coding DNA (cDNA) of 2.357 kb. The protein has a weight of 52 kilodaltons (kDa) and contains 491 amino acids (aa) (46). The protein lacks a cellular export signal and also contains 6 cysteine residues, so it has been suggested a structure of the same as zinc finger.

*In silico*, up to 13 messengers of different NAMPT RNAs (mRNA) are predicted by alternative splicing mechanisms, of which only four have been found at the biological level. Of the four, only the first messenger translating into the 491 amino acid protein is able to make the conversion to its enzymatic product NMN.

NAMPT is expressed in all tissues, with higher levels in bone marrow, liver, and muscle fiber cells, where the energy intake is greater.

The extracellular form of NAMPT, PBEF, or Visfatin is known as extracellular NAMPT (eNAMPT, as opposed to intracellular NAMPT, iNAMPT), an adipocytokine that is expressed in visceral fat tissue and whose circulating levels correlate with obesity (43). The extracellular role of NAMPT is unknown and could have a function of activation or silencing in signaling pathways within the cell other than those related to its enzymatic function such as NAMPT.

As a limiting enzyme of the pathway which plays a key role in the maintenance of intracellular NAD+, NAMPT could be an oncogene contributing to the onset, progression and relapse of cancer (47).

## NAMPT in Cancer

NAMPT is overexpressed in a broad range of solid tumors including colorectal, ovarian, breast, gastric, prostate, well-differentiated thyroid cancers, melanoma, gliomas, and endometrial carcinomas (48–51). Clinically, higher NAMPT expression is associated with worse prognosis correlating with tumor growth, metastases and cellular dedifferentiation in melanoma (52, 53). High levels of NAMPT have been found in hematological malignancies such as diffuse large B-cell lymphoma, Hodgkin's lymphoma, follicular B-cell lymphoma, and peripheral T-cell lymphoma. In these tumors, it associates to a more aggressive malignant lymphoma phenotype (54).

Besides, NAMPT levels have been associated to increased chemoresistance to certain therapeutic agents such as doxorubicin, paclitaxel, etoposide, fluorouracil, and phenylethyl isothiocyanate (55, 56).

Many studies have shown that NAD^+^ depletion by NAMPT inhibition causes cell death through apoptosis. Many pro-apoptotic proteins were found activated when NAMPT is inhibited in leukemias, multiple myeloma, breast cancer, and lymphoma cells (57–64). It has been found that NAMPT inhibition-mediated apoptosis requires functional apoptotic machinery because blocking apoptosis with several factors such as: L-type calcium channels with verapamil or nimodipine, capase 3 with Z-Asp-Glu Val-Asp-fluoromethylketone, capase 9 with Z-Leu-Glu-His-Asp-fluoromethylketone, or the mitochondrial permeability transition with bongkrekic acid blocks the effect of NAMPT inhibition-mediated apoptosis (62, 64).

Three NAMPT inhibitors (APO866/FK866, GMX1778, and GMX1777) entered clinical trials and completed phase I, however, further evaluation was discontinued primarily due to dose-limiting toxicities (ClinicalTrials.gov identifiers: NCT00457574, NCT00724841, NCT00432107, NCT00435084, NCT00431912).

More enzymes of the salvage pathway have been suggested to be potential therapeutic targets in cancer. Mitochondrial NMNAT3 knockdown had minimal effect over mitochondrial NAD+ levels (37, 65, 66). On the other hand, NMNAT2 cytosolic inhibition decreased mitochondrial NAD+ levels, suggesting that NAD+ in the mitochondrial is partially supported by NAD+ intake from the cytosol (65). However, shortly after it was found that NAMPT inhibition had no effect on mitochondrial NAD^+^ pool, discarding the previous theory, and highlighting the role of NAMPT as the main potential target of the pathway in cancer by depleting NAD^+^ pool (40). NAMPT inhibition seems to be particularly effective over cells harboring naturally high glycolysis (67).

## Causal Role of NAD+ in Glioma Stem-Like Cells Transformation

NAD^+^ is an important cofactor for cells requiring high energetics demands and helps to maintain a proficient neural function. Indeed, the increase in NAD^+^ levels shown as Nicotinamide nucleotide transhydrogenase (NNT) and Nicotinamide nucleotide adenylyltransferase 3 (NMNAT3) delays senescence in mesenchymal stem cells (68). In relation, it is known that NAMPT and NAD^+^ biosynthesis decreases with age in the hippocampus (69). Also, NAMPT downregulation and NAD^+^ depletion reduces pool and proliferation of Neural stem progenitor cells (NSPCs) *in vivo* (70). Increased NR decreases senescence in both neural and melanocyte stem cells by improving mitochondrial function relying on SIRT1 function (68). It has been reported that NAD^+^ replenishment reduces the severity of Ataxia telangiectasia (A-T) neuropathology, normalizing neuro-muscular function, extending lifespan in animal models, and delaying memory loss (71). Moreover, intracellular NAD^+^ levels stimulates neural DNA repair through PARP proteins and improves mitochondrial activity via mitophagy (71).

Cancer cells exhibit a dependency on metabolic pathways regulated by NAD^+^. Nonetheless, the regulatory network interfacing with signal transduction remains poorly understood in GBM and HGG. NAMPT downregulation reduces *in vivo* tumorigenicity (72). Indeed, RNA-seq reveals the transcription factor E2F2 in the center of an NAD^+^-dependent transcriptional network, required to self-renewal maintenance in GSCs. Interestingly, downstream to E2F2 we can find members of the inhibitor of differentiation (ID) helix-loop-helix gene family (72).

NAMPT and NAD^+^ levels also mediate GSCs radio-resistance. NAD^+^ pool decreases with aging and their levels are critical in cell bioenergetics and adaptive stress responses (73).

Another important consequence driven by NAD^+^ supply is the epigenetic reprogramming in tumors. Nicotinamide N-methyltransferase (NNMT) is a critical node in methyl donor metabolism and is markedly upregulated in GBM (15). NNMT is also overexpressed in mesenchymal stem cells. Increases in NNMT lead to a decrease in S-Adenosyl methionine (SAM), a methyl donor generated from methionine. GSCs show a decrease in methionine and SAM, thereby decreased levels of Nicotinamide (15). However, GSCs show notably increased NAD^+^ levels and the dramatic hypomethylation state in GBM, leading tumors to shift toward mesenchymal phenotype and accelerated growth, where NAMPT is particularly overexpressed (15). Targeting NNMT decreases cellular proliferation and diminishes methyl donor availability, thus decreasing methionine levels. This fact also leads to the induction of decreasing unmethylated cytosine levels, increased DNA methyltransferases and induced expression of *de novo* methylases like DNMT1 and DNMT3A (15).

NAMPT is ultimately required for G1/S progression of the Neural Stem Progenitor Cells (NSPC) cell cycle (69). Indeed, NAMPT is crucial for oligodendrocytic lineage fate decisions through an overlapping mechanism mediated by Sirt1 and Sirt2. Reported studies on this topic proved that NAMPT downregulation *in vivo* leads to a decreased NSPC-mediated oligodendrogenesis (69).

## Role of Sirtuins in Glioma Progression

Sirtuins are NAD^+^ dependent deacetylases that regulate numerous cellular processes including aging, cell cycle, metabolism, DNA repair, and survival under stress conditions. Nonetheless, the role of Sirtuins in glioma remains unclear, with some studies counteracting others. Whether they act as an oncogene or tumor suppressor in Glioma progression is unclear, but their contribution seems to be correlated with tumor heterogeneity.

On one hand, SIRT1 is characterized by some authors as a promoter factor in tumorigenesis of human glioma. The role of SIRT1 in glioma may be related with PTEN/PI3K/Akt axis promoting tumorigenicity (74).

On the other hand, some studies point that SIRT2 is required for GSCs proliferation arrest, highlighting a potential tumor suppressive effect over GSCs (75). Nonetheless, Funato et al. proposed a mechanism where SIRT2-mediated inactivation of p73 is required for GBM tumorigenicity (76).

Accordingly, Li et al. found that SIRT2 expression is markedly down-regulated in human glioma. Indeed, its expression decreases cell growth and colony formation via apoptosis. Mechanistically, they claim that mIR-21 is essential for the functions of SIRT2 in these tumors. SIRT2 specifically deacetylases p65 to decrease mIR-21 expression (77). They conclude in their study that SIRT2 suppresses glioma cell growth through targeting NF-kB-mIR-21 axis (77).

Other Sirtuins like SIRT6 suppresses cell proliferation, migration, and invasion via inhibition of oxidative stress through NOTCH3 (78) or inhibition of the activation of the JAK2/STAT3 pathway in glioma (79).

Mechanistically, miR-33a targets SIRT6 and promotes tumor development in human glioma by regulating its expression (80).

## Replication Stress: Role of PARP Proteins in Glioma Progression

GSCs are Mismatch repair-proficient (MMR-proficient) cells. TMZ-resistant GSCs are either O^6^-methylguanine-DNA methyltransferase (MGMT) active or displaying low proliferation rate. MMR-proficiency leads GSCs to select networks to effectively repair DNA driven by TMZ and radiotherapy (81). GSCs are also highly radioresistant. This radio resistance was originally overcome for the first time through CHK1 and CHK2 inhibition (82).

**Figure 3:**
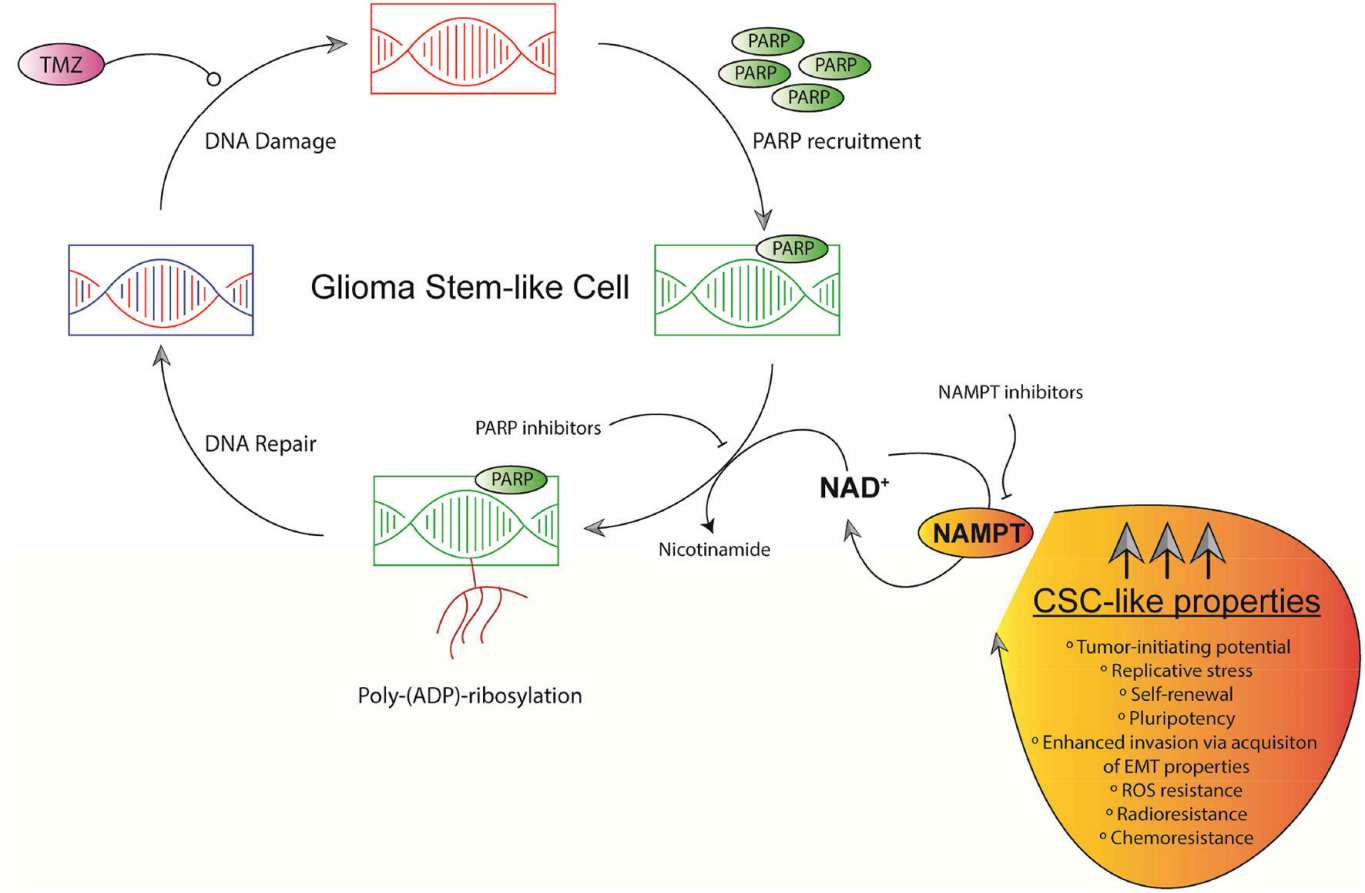
Schematic representation of GSCs' replication stress and NAD^+^-dependent DNA repair mechanisms. TMZ induces DNA damage in chromosomal DNA in GSCs. PARPs-based DNA repair defines chemosensitivity. PARPs efficacy relies on NAD^+^ levels mainly controlled by NAMPT. NAMPT dually governs NAD^+^ production and maintain CSC-like properties in GSCs.

NAD^+^ plays a key role enhancing Base excision repair (BER) pathway through Poly(ADP-ribose) polymerases (PARPs) (83). PARP1 expression is increased in GBM at both mRNA and protein level. Increased PARP1 levels show a positive correlation with increasing tumor grades in Gliomas. PARP1 is ultimately essential for DNA repair during TMZ-based treatment and radiation therapy (Figure 3) (83). PARP1 expression is associated with TP53 and ataxia telangiectasia—Rad3 related kinase (ATR) mutations (84). GSCs display elevated basal levels of activated ATR and CHK1 along with increased replication stress (RS) expression markers including foci marked with the single-stranded DNA binding protein, replication protein A, DNA damage markers γH2AX and 53BP1 (82). PARP1 is mainly overexpressed in Proneural and Classical GBM subtypes. PARP1 overexpression decreases OS in patients with classical type GBM (84). GSCs display a heightened DNA damage response (DDR) with a markedly enhanced replication stress. In order to overcome radiation resistance relying on G_2_-M activation, combined inhibition of ATR and PARP inhibitors proved to be effective (85–87). Since chemical inhibition of PARP1 through Olaparib also impairs BER, it significantly enhances TMZ-induced damage (Figure 3), exerting synergistic anti-tumor effects in GSCs lines.

NAD^+^ availability is substantially decreased in IDH1-mutants', thus, PARP1-associated DNA repair pathway is compromised. Targeting DNA repair pathway through PARP inhibition sensitizes IDH1-mutated glioma cells to TMZ in combinational therapy (83).

When radio- or chemo-driven DNA damage occurs, PARP1 and PARP2 enzymes are recruited as a systemic damage response to bind to ssDNA breaks and transduce signals within the DDR pathways (82). Once the bond to ssDNA breaks, damaged cells activate PARP1/2 to post-translationally modify themselves together with proteins by synthesizing negatively charged poly(ADP-ribose) chains.

Poly-ADP ribosylation recruits proteins involved in ssDNA break repair, for example XRCC1 modifying chromatin structure. Regulating fork stabilization and restart, PARP proteins work on DNA replication, RS response ligation of lagging strand Okazaki fragments, elongation velocity, and homologous recombination repair of stalled DNA replication forks (88–92).

PARP “trapping” through PARP inhibitors leads to loss of auto-PARylation, consequently facilitating removal of PARP protein from DNA. ATR inhibitors decreases GSCs formation *in vitro*. PARP inhibitors plus ATR inhibitors dramatically decrease GSC formation and sensitize to radiotherapy. This phenotype is greater in GSCs-CD133^+^ cells compared to bulk population (82, 93, 94).

To note, given the well-characterized immunosuppressive tumor microenvironment associated with GBM, ATR treatment may present two weapons against this disease: targeting the addiction to the DDR pathway and reinvigorating T cells to attack GBM cells following radiotherapy (87, 95).

One of the major limitations establishing PARP or ATR inhibitors as a standard therapy is the BBB, which means an ineffective drug penetration into the central nervous system.

## NAMPT as a Dedifferentiation-Inducer Gene in Glioma

NAMPT is markedly overexpressed in HGG and GBM tumors, correlating with tumor grade and able to predict patients' prognosis. Ectopic overexpression of NAMPT in Glioma cell lines increases tumorigenic properties controlling stem cell pathways and enriching the GSCs population (Figure 3) (96). Given the infiltrative nature of GBM portraying high plasticity, many studies have tried to focus on identifying key factors governing self-renewal properties driving to tumor relapses. Gujar et al. demonstrated that a NAD^+^ transcriptional network governs self-renewal properties in GSCs and radiation resistance in GBM (72). NAMPT expression, indeed, correlates with high levels of NANOG as a final effector of pluripotency and stem cell-like properties, CD133^+^, and GSCs in primary GBM tumors. NAMPT is also a key factor inducing cancer stem-like pathways in glioma cells. NAMPT also increases number of *de novo* GSCs formation (96). Moreover, NAMPT is also a key factor inducing cancer stem pathway effectors in colon cancer tumors. In colon cancer, this phenotype is mediated by PARP and SIRT1. NAMPT also increases the number of tumorspheres *in vitro* in colon cancer cells lines, correlating with important endpoints of CSC pathways activation (97).

## Current Therapies on Glioma Treatment

Sixty gray (Gy) dose of radiotherapy following the maximum safe surgical resection provides the highest benefits regarding GBM median survival (98). TMZ addition after radiotherapy or in concurrence is the only regimen markedly improving the patients' overall survival (OS) (99, 100). The major relevant biomarker predicting response to TMZ treatment is MGMT (O^6^- methylguanine-DNA methyltransferase), a gene involved in DNA-repair (101). Silencing MGMT expression through promoter methylation impairs the ability to repair TMZ-driven DNA damage, decreasing tumor cell survival (102).

Since GBM tumors are often overexpressing numerous angiogenic effectors, Bevacizumab, a humanized antibody binding to vascular endothelial growth factor A (VEGF-A) is effective in impairing tumor angiogenesis. Bevacizumab is actually the only FDA approved agent for recurrent GBM (103, 104). Irinotecan (topoisomerase I inhibitor) in combination with Bevacizumab proved an increase in OS from 4.1 to 9.2 months in a phase II clinical trial (104). 6 and 12 months' survival rates were 77 and 31%, respectively (105, 106). Bevacizumab has been also tested in phase III trials for newly diagnosed GBM. However, there was no effect on patients' OS. TMZ administration in concurrence with Bevacizumab on newly diagnosed GBM is being tested (NCT00943826 and NCT00884741) (106, 107).

Proven that most of the current attempts on therapies seem to be insufficient to achieve relevant outcomes regarding OS, targeting NAD^+^ through NAMPT as the central core axis of GSCs energetics maintenance could represent a ground-breaking therapy approach.

## Targeting NAMPT on Glioma Cancer Stem-Like Cells for Glioma Control

NAMPT is markedly overexpressed in HGG and GBM. NAMPT overexpression is correlated with patient survival (96). NAMPT downregulation triggers a markedly decrease in *in vivo* tumorigenicity and induces a decrease in GSCs self-renewal capacity (72). Indeed, first generations of tumorspheres *in vitro* are particularly sensitive to NAMPT inhibitors, particularly tumorspheres with high levels of NAMPT expression. Unlike glioma cells, NAMPT inhibitors will ultimately target cells in an active cell cycle under replicative stress, two main hallmarks of GSCs (96). This fact potentially ensures that anti-NAMPT therapies in either monotherapy or in combination with TMZ or PARP inhibitors will be ultimately effective. Targeting proliferating cells relying on NAD+ may have effects over GSCs in two main ways: first, by suppressing self-renewal properties based on NAD+ pool restoration and recycling and second, by taking down ADP-ribosylation required for DNA repair process through PARP recruitment.

## Conclusion and Perspectives

Gliomas are the most prevalent primary brain cancer in adults and include a broad category of tumors including astrocytoma, oligodendroglioma, and GBM. Regardless of tumor aggressiveness, malignancy, and infiltration, these glia-derived tumors rarely exceed a median survival time of 12–14 months. Driven by the infiltrative nature of these tumors, the clinical approach is difficult and relapses often occur with fatal consequences. These unsuccessful attempts to control glioma's fate have fostered research looking for more effective therapies.

(GSCs) are a small subset of CD133^+^ cells with self-renewal properties and capable of initiating new tumors contributing to Glioma progression, maintenance, hierarchy and complexity. GSCs are highly resistant to chemo and radiotherapy. These cells are believed to be responsible for tumor relapses and patients' fatal outcome after developing a recurrent GBM or High Grade Glioma (HGG). GSCs are cells under replicative stress with high demands on NAD^+^ supply to repair DNA, maintain self-renewal capacity and to induce tumor plasticity. NAD^+^ feeds Poly-ADP polymerases (PARP) and NAD^+^-dependent deacetylases (SIRTUINS) contributing to GSC phenotype. Ablation of NAMPT showed to impair Schwann cell differentiation program associated to reduced NAD^+^ levels, counteracting the phenotype found in tumor cells but reinforcing the idea that an adequate NAD^+^ level is required for a fine tuned balance on cell-fate definition toward differentiation programs (108). This energetic core axis is mainly controlled by the rate-limiting enzyme nicotinamide phosphoribosyltransferase (NAMPT), an important oncogene contributing to tumor dedifferentiation. Supporting these conclusions, Jung et al. also highlight the importance of the epigenetic reprogramming on GSC, supported by a high expression of NNMT together with NAMPT counteracting with a hypomethylation state, preferentially occurring within mesenchymal GBM subtype, where NAMPT shows its maximum expression profile (96). They raise the idea that one causal role of methionine depletion, an important upstream methyl donor, is to ultimately drive GBMs to evolve to mesenchymal subtype, where NAMPT plays a key role promoting tumor growth and dedifferentiating tumors (15).

We have queried public datasets to dissect these proposed mechanisms at a single cell level. For that, we have analyzed a single-cell RNA-Seq dataset from Darmanis et al. (109) performing comparative transcriptomic analysis of neoplastic and stromal cells within and proximal to primary GBMs (109).

**Figure 4:**
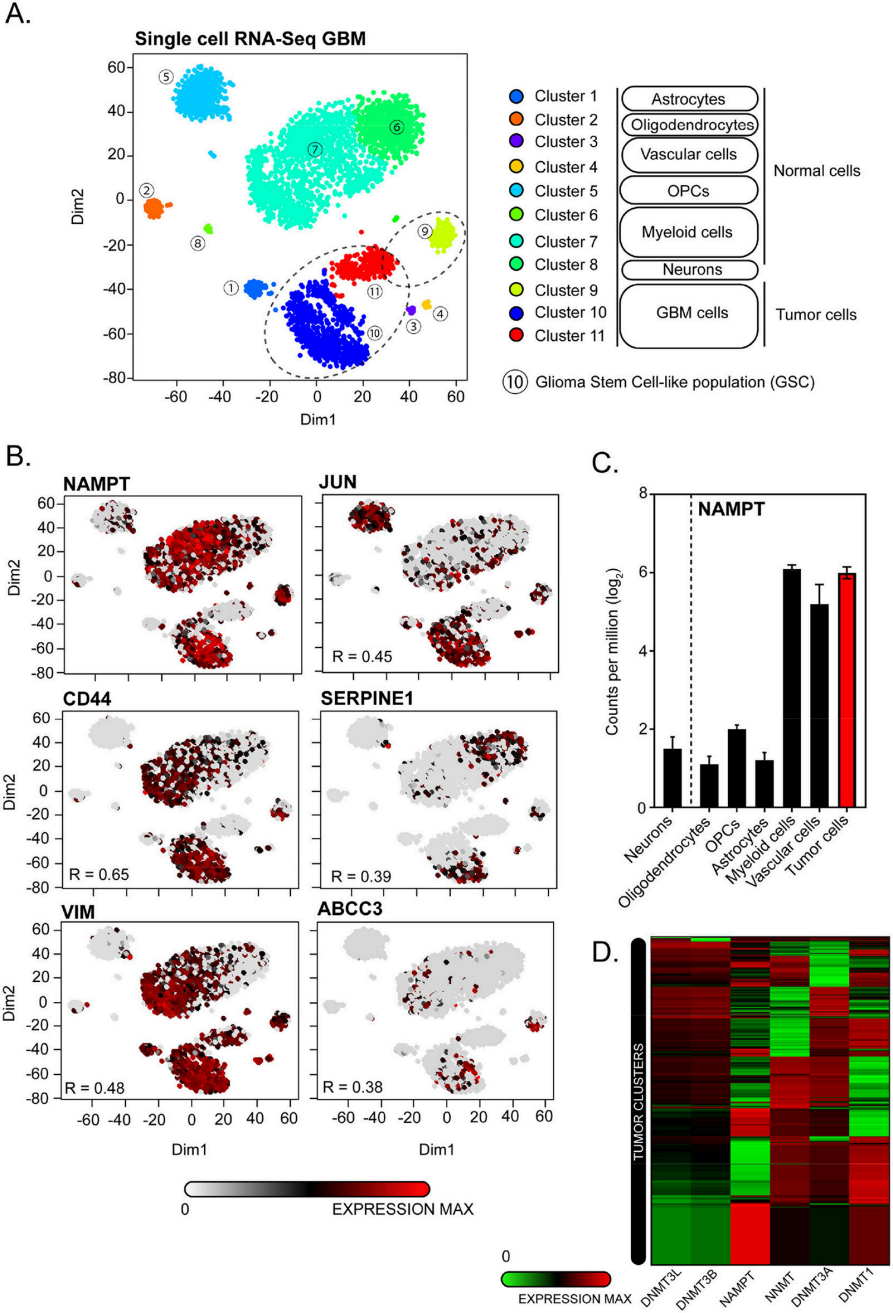
Single-cell transcriptomic analysis of GSE84465 for *NAMPT* expression in GBM. **(A)** 3,589 cells in a cohort of 4 patients are categorized by cell identity (Normal vs. Tumoral) and clusters based on differences on transcriptomic profiles based on heterogenic distribution on dimensions 1 and 2 of t-distributed stochastic neighbor embedding (tSNE). Three tumors clusters are highlighted on dashed circles. Cluster number 10 is transcriptionally allotted to GSC-cells subset. **(B)** Single-cell transcriptomic distribution for GSC-related markers expression in correlation to *NAMPT* expression. **(C)** *NAMPT* expression analysis classified by different cell identities from normal and tumor tissue. **(D)** Supervised hierarchical clustering of *NAMPT, NNMT, DNMT1, DNMT3A, DNMT3B*, and *DNMT3L* mRNA expression in tumor cell clusters (9, 10, and 11).

In this dataset, three clusters out of 11 are identified as tumor clusters (Figure 4A). We found that NAMPT is particularly expressed in vascular, myeloid, and tumor cells (Figure 4C). Within tumor cells, NAMPT is particularly overexpressed in cluster 9 and 10 but not in cluster 11 (Figure 4B), which is a cluster with a pro-neural profiling and therefore with low NAMPT levels, in accordance with the results showed by Lucena et al. and Jung et al. Cluster 10 is classified as GSCs subset, where NAMPT is highly overexpressed. According to the high contribution to data dimensionality of XY axis in the t-distributed stochastic neighbor embedding (tSNE), the GSC cluster shows a high heterogeneity, reinforcing the idea that GSCs rely on a high diversity in terms of molecular-profiling, sustaining an ever-changing identity contributing to tumor plasticity. With the aim of confirming the expression of factors that induce cell stemness and pluripotency in correlation to NAMPT levels, we observed a clear transcriptional activation of JUN, CD44, SERPINE1, VIM, and ABCC3 putative markers of GSCs maintenance as previously described by Lucena et al. within the tumor clusters.

In addition, NAMPT expression negatively correlates with *de novo* methyltransferases DNMT1, DNMT3A, DNMT3B, and DNMT3L, supporting the hypothesis raised by Jung et al. where the poor outcome of GBMs is related to a hypomethylation state driven by overexpression of NAMPT controlling NAD^+^ pool since mesenchymal GSCs rapidly consume NAM to support NAD^+^ utilization and sustain DNA hypomethylation (Figure 4D) (15).

Altogether, targeting GSCs depicts a new frontier in Glioma therapy; hence NAMPT could represent a key regulator for GSCs maintenance. Its inhibition may attenuate GSCs properties by decreasing NAD^+^ supply, consequently contributing a better outcome together with current therapies for Glioma control.

## Author Contributions

AL-C and AC conceived and designed the project. AL-C, MU, and LEN collected, processed, and analyzed experimental data. AL-C and AC wrote the manuscript. All authors discussed the results.

### Conflict of Interest Statement

The authors declare that the research was conducted in the absence of any commercial or financial relationships that could be construed as a potential conflict of interest.

## References

[1] Le Rhun E Taillibert S Chamberlain MC. Anaplastic glioma: current treatment and management. Expert Rev Neurother. (2015) 15:601–20. 10.1586/14737175.2015.1042455 25936680

[2] Zeng T Cui D Gao L. Glioma: an overview of current classifications, characteristics, molecular biology, and target therapies. Front Biosci. (2015) 20:1104–15. 10.2741/4362 25961548

[3] Morokoff A Ng W Gogos A Kaye AH. Molecular subtypes, stem cells and heterogeneity: implications for personalised therapy in glioma. J Clin Neurosci. (2015) 22:1219–26. 10.1016/j.jocn.2015.02.008 25957782

[4] Reardon DA Wen PY. Glioma in 2014: unravelling tumour heterogeneity-implications for therapy. Nat Rev Clin Oncol. (2015) 12:69–70. 10.1038/nrclinonc.2014.223 25560529

[5] Singh SK Hawkins C Clarke ID Squire JA Bayani J Hide T. Identification of human brain tumour initiating cells. Nature. (2004) 432:396–401. 10.1038/nature03128 15549107

[6] Louis DN Perry A Reifenberger G von Deimling A Figarella-Branger D Cavenee WK. The 2016 world health organization classification of tumors of the central nervous system: a summary. Acta Neuropathol. (2016) 131:803–20. 10.1007/s00401-016-1545-1 27157931

[7] Karsy M Gelbman M Shah P Balumbu O Moy F Arslan E. Established and emerging variants of glioblastoma multiforme: review of morphological and molecular features. Folia Neuropathol. (2012) 50:301–21. 10.5114/fn.2012.32361 23319187

[8] Davis ME. Glioblastoma: overview of disease and treatment. Clin J Oncol Nurs. (2016) 20:S2–8. 10.1188/16.CJON.S1.2-8 27668386 PMC5123811

[9] Brennan CW Verhaak RG McKenna A Campos B Noushmehr H Salama SR. The somatic genomic landscape of glioblastoma. Cell. (2013) 155:462–77. 10.1016/j.cell.2013.09.034 24120142 PMC3910500

[10] Verhaak RG Hoadley KA Purdom E Wang V Qi Y Wilkerson MD. Integrated genomic analysis identifies clinically relevant subtypes of glioblastoma characterized by abnormalities in PDGFRA, IDH1, EGFR, and NF1. Cancer Cell. (2010) 17:98–110. 10.1016/j.ccr.2009.12.020 20129251 PMC2818769

[11] Cancer Genome Atlas Research Network Comprehensive genomic characterization defines human glioblastoma genes and core pathways. Nature. (2008) 455:1061–8. 10.1038/nature07385 18772890 PMC2671642

[12] Parsons DW Jones S Zhang X Lin JC Leary RJ Angenendt P. An integrated genomic analysis of human glioblastoma multiforme. Science. (2008) 321:1807–12. 10.1126/science.1164382 18772396 PMC2820389

[13] Maus A Peters GJ Glutamate and alpha-ketoglutarate: key players in glioma metabolism. Amino Acids. (2017) 49:21–32. 10.1007/s00726-016-2342-9 27752843 PMC5241329

[14] Mergenthaler P Lindauer U Dienel GA Meisel A. Sugar for the brain: the role of glucose in physiological and pathological brain function. Trends Neurosci. (2013) 36:587–97. 10.1016/j.tins.2013.07.001 23968694 PMC3900881

[15] Jung J Kim LJY Wang X Wu Q Sanvoranart T Hubert CG. Nicotinamide metabolism regulates glioblastoma stem cell maintenance. JCI Insight. (2017) 2:e90019. 10.1172/jci.insight.90019 28515364 PMC5436539

[16] Alcantara Llaguno SR Parada LF. Cell of origin of glioma: biological and clinical implications. Br J Cancer. (2016) 115:1445–50. 10.1038/bjc.2016.354 27832665 PMC5155355

[17] Visvader JE. Cells of origin in cancer. Nature. (2011) 469:314–22. 10.1038/nature09781 21248838

[18] Kim J Lee IH Cho HJ Park CK Jung YS Kim Y. Spatiotemporal evolution of the primary glioblastoma genome. Cancer Cell. (2015) 28:318–28. 10.1016/j.ccell.2015.07.013 26373279

[19] Shao F Liu C. Revisit the candidacy of brain cell types as the cell(s) of origin for human high-grade glioma. Front Mol Neurosci. (2018) 11:48. 10.3389/fnmol.2018.00048 29515370 PMC5826356

[20] Patel AP Tirosh I Trombetta JJ Shalek AK Gillespie SM Wakimoto H. Single-cell RNA-seq highlights intratumoral heterogeneity in primary glioblastoma. Science. (2014) 344:1396–401. 10.1126/science.1254257 24925914 PMC4123637

[21] Liu T Xu H Huang M Ma W Saxena D Lustig RA. Circulating glioma cells exhibit stem cell-like properties. Cancer Res. (2018) 78:6632–42. 10.1158/0008-5472.CAN-18-0650 30322863 PMC6497085

[22] Belenky P Bogan KL Brenner C. NAD+ metabolism in health and disease. Trends Biochem Sci. (2007) 32:12–9. 10.1016/j.tibs.2006.11.006 17161604

[23] Faraone-Mennella MR. A new facet of ADP-ribosylation reactions: SIRTs and PARPs interplay. Front Biosci. (2015) 20:458–73. 10.2741/4319 25553461

[24] Koch-Nolte F Fischer S Haag F Ziegler M. Compartmentation of NAD+-dependent signalling. FEBS Lett. (2011) 585:1651–6. 10.1016/j.febslet.2011.03.045 21443875

[25] Dolle C Skoge RH Vanlinden MR Ziegler M. NAD biosynthesis in humans–enzymes, metabolites and therapeutic aspects. Curr Top Med Chem. (2013) 13:2907–17. 10.2174/15680266113136660206 24171775

[26] Houtkooper RH Canto C Wanders RJ Auwerx J. The secret life of NAD+: an old metabolite controlling new metabolic signaling pathways. Endocr Rev. (2010) 31:194–223. 10.1210/er.2009-0026 20007326 PMC2852209

[27] Magni G Orsomando G Raffelli N Ruggieri S. Enzymology of mammalian NAD metabolism in health and disease. Front Biosci. (2008) 13:6135–54. 10.2741/3143 18508649

[28] Revollo JR Korner A Mills KF Satoh A Wang T Garten A. Nampt/PBEF/Visfatin regulates insulin secretion in beta cells as a systemic NAD biosynthetic enzyme. Cell Metab. (2007) 6:363–75. 10.1016/j.cmet.2007.09.003 17983582 PMC2098698

[29] Rongvaux A Shea RJ Mulks MH Gigot D Urbain J Leo O. Pre-B-cell colony-enhancing factor, whose expression is up-regulated in activated lymphocytes, is a nicotinamide phosphoribosyltransferase, a cytosolic enzyme involved in NAD biosynthesis. Eur J Immunol. (2002) 32:3225–34. 10.1002/1521-4141(200211)32:11and<3225::AID-IMMU3225and>3.0.CO;2-L 12555668

[30] Magni G Amici A Emanuelli M Raffaelli N Ruggieri S. Enzymology of NAD+ synthesis. Adv Enzymol Relat Areas Mol Biol. (1999) 73:135–82, xi. 10.1002/9780470123195.ch5 10218108

[31] Bender DA. Biochemistry of tryptophan in health and disease. Mol Aspects Med. (1983) 6:101–97. 10.1016/0098-2997(83)90005-5 6371429

[32] Bender DA Olufunwa R. Utilization of tryptophan, nicotinamide and nicotinic acid as precursors for nicotinamide nucleotide synthesis in isolated rat liver cells. Br J Nutr. (1988) 59:279–87. 10.1079/BJN19880035 2965917

[33] Berger F Lau C Dahlmann M Ziegler M. Subcellular compartmentation and differential catalytic properties of the three human nicotinamide mononucleotide adenylyltransferase isoforms. J Biol Chem. (2005) 280:36334–41. 10.1074/jbc.M508660200 16118205

[34] Emanuelli M Carnevali F Saccucci F Pierella F Amici A Raffaelli N. Molecular cloning, chromosomal localization, tissue mRNA levels, bacterial expression, and enzymatic properties of human NMN adenylyltransferase. J Biol Chem. (2001) 276:406–12. 10.1074/jbc.M008700200 11027696

[35] Lau C Niere M Ziegler M. The NMN/NaMN adenylyltransferase (NMNAT) protein family. Front Biosci. (2009) 14:410–31. 10.2741/3252 19273075

[36] Warburg O. On the origin of cancer cells. Science. (1956) 123:309–14. 10.1126/science.123.3191.309 13298683

[37] Felici R Lapucci A Ramazzotti M Chiarugi A. Insight into molecular and functional properties of NMNAT3 reveals new hints of NAD homeostasis within human mitochondria. PLoS ONE. (2013) 8:e76938. 10.1371/annotation/f5e6107f-a911-4c15-a881-7cb7e4946ff6 24155910 PMC3796565

[38] Hikosaka K Ikutani M Shito M Kazuma K Gulshan M Nagai Y. Deficiency of nicotinamide mononucleotide adenylyltransferase 3 (nmnat3) causes hemolytic anemia by altering the glycolytic flow in mature erythrocytes. J Biol Chem. (2014) 289:14796–811. 10.1074/jbc.M114.554378 24739386 PMC4031534

[39] Zhang X Kurnasov OV Karthikeyan S Grishin NV Osterman AL Zhang H. Structural characterization of a human cytosolic NMN/NaMN adenylyltransferase and implication in human NAD biosynthesis. J Biol Chem. (2003) 278:13503–11. 10.1074/jbc.M300073200 12574164

[40] Pittelli M Formentini L Faraco G Lapucci A Rapizzi E Cialdai F. Inhibition of nicotinamide phosphoribosyltransferase: cellular bioenergetics reveals a mitochondrial insensitive NAD pool. J Biol Chem. (2010) 285:34106–14. 10.1074/jbc.M110.136739 20724478 PMC2962509

[41] Yang H Yang T Baur JA Perez E Matsui T Carmona JJ. Nutrient-sensitive mitochondrial NAD+ levels dictate cell survival. Cell. (2007) 130:1095–107. 10.1016/j.cell.2007.07.035 17889652 PMC3366687

[42] McKenna MC Waagepetersen HS Schousboe A Sonnewald U. Neuronal and astrocytic shuttle mechanisms for cytosolic-mitochondrial transfer of reducing equivalents: current evidence and pharmacological tools. Biochem Pharmacol. (2006) 71:399–407. 10.1016/j.bcp.2005.10.011 16368075

[43] Gatenby RA Gillies RJ. Why do cancers have high aerobic glycolysis? Nat Rev Cancer. (2004) 4:891–9. 10.1038/nrc1478 15516961

[44] Rongvaux A Andris F Van Gool F Leo O. Reconstructing eukaryotic NAD metabolism. Bioessays. (2003) 25:683–90. 10.1002/bies.10297 12815723

[45] Yalowitz JA Xiao S Biju MP Antony AC Cummings OW Deeg MA. Characterization of human brain nicotinamide 5'-mononucleotide adenylyltransferase-2 and expression in human pancreas. Biochem J. (2004) 377:317–26. 10.1042/bj20030518 14516279 PMC1223862

[46] Zhang T Berrocal JG Frizzell KM Gamble MJ DuMond ME Krishnakumar R. Enzymes in the NAD+ salvage pathway regulate SIRT1 activity at target gene promoters. J Biol Chem. (2009) 284:20408–17. 10.1074/jbc.M109.016469 19478080 PMC2740465

[47] Brooks GA. Lactate production under fully aerobic conditions: the lactate shuttle during rest and exercise. Fed Proc. (1986) 45:2924–9. 3536591

[48] Sawicka-Gutaj N Waligorska-Stachura J Andrusiewicz M Biczysko M Sowinski J Skrobisz J. Nicotinamide phosphorybosiltransferase overexpression in thyroid malignancies and its correlation with tumor stage and with survivin/survivin DEx3 expression. Tumour Biol. (2015) 36:7859–63. 10.1007/s13277-015-3506-z 25946974 PMC4605962

[49] Shackelford RE Mayhall K Maxwell NM Kandil E Coppola D. Nicotinamide phosphoribosyltransferase in malignancy: a review. Genes Cancer. (2013) 4:447–56. 10.1177/1947601913507576 24386506 PMC3877665

[50] Vora M Ansari J Shanti RM Veillon D Cotelingam J Coppola D. Increased Nicotinamide phosphoribosyltransferase in rhabdomyosarcomas and leiomyosarcomas compared to skeletal and smooth muscle tissue. Anticancer Res. (2016) 36:503–7. 26851003 PMC7771545

[51] Wang S Xing Z Vosler PS Yin H Li W Zhang F. Cellular NAD replenishment confers marked neuroprotection against ischemic cell death: role of enhanced DNA repair. Stroke. (2008) 39:2587–95. 10.1161/STROKEAHA.107.509158 18617666 PMC2743302

[52] Maldi E Travelli C Caldarelli A Agazzone N Cintura S Galli U. Nicotinamide phosphoribosyltransferase (NAMPT) is over-expressed in melanoma lesions. Pigment Cell Melanoma Res. (2013) 26:144–6. 10.1111/pcmr.12037 23051650

[53] Reddy PS Umesh S Thota B Tandon A Pandey P Hegde AS. PBEF1/NAmPRTase/Visfatin: a potential malignant astrocytoma/glioblastoma serum marker with prognostic value. Cancer Biol Ther. (2008) 7:663–8. 10.4161/cbt.7.5.5663 18728403

[54] Olesen UH Hastrup N Sehested M. Expression patterns of nicotinamide phosphoribosyltransferase and nicotinic acid phosphoribosyltransferase in human malignant lymphomas. APMIS. (2011) 119:296–303. 10.1111/j.1600-0463.2011.02733.x 21492230

[55] Bi TQ Che XM Liao XH Zhang DJ Long HL Li HJ. Overexpression of Nampt in gastric cancer and chemopotentiating effects of the Nampt inhibitor FK866 in combination with fluorouracil. Oncol Rep. (2011) 26:1251–7. 10.3892/or.2011.1378 21743967

[56] Folgueira MA Carraro DM Brentani H Patrao DF Barbosa EM Netto MM. Gene expression profile associated with response to doxorubicin-based therapy in breast cancer. Clin Cancer Res. (2005) 11:7434–43. 10.1158/1078-0432.CCR-04-0548 16243817

[57] Cagnetta A Cea M Calimeri T Acharya C Fulciniti M Tai YT. Intracellular NAD(+) depletion enhances bortezomib-induced anti-myeloma activity. Blood. (2013) 122:1243–55. 10.1182/blood-2013-02-483511 23823317 PMC3744991

[58] Cea M Cagnetta A Acharya C Acharya P Tai YT Yang C. Dual NAMPT and BTK targeting leads to synergistic killing of waldenstrom macroglobulinemia cells regardless of MYD88 and CXCR4 somatic mutation status. Clin Cancer Res. (2016) 22:6099–109. 10.1158/1078-0432.CCR-16-0630 27287071 PMC5771267

[59] Gehrke I Bouchard ED Beiggi S Poeppl AG Johnston JB Gibson SB. On-target effect of FK866, a nicotinamide phosphoribosyl transferase inhibitor, by apoptosis-mediated death in chronic lymphocytic leukemia cells. Clin Cancer Res. (2014) 20:4861–72. 10.1158/1078-0432.CCR-14-0624 25172933

[60] Hansen CM Hansen D Holm PK Larsson R Binderup L. Cyanoguanidine CHS 828 induces programmed cell death with apoptotic features in human breast cancer cells *in vitro*. Anticancer Res. (2000) 20:4211–20. 11205250

[61] Martinsson P Ekelund S Nygren P Larsson R. The combination of the antitumoural pyridyl cyanoguanidine CHS 828 and etoposide *in vitro*–from cytotoxic synergy to complete inhibition of apoptosis. Br J Pharmacol. (2002) 137:568–73. 10.1038/sj.bjp.0704888 12359640 PMC1573513

[62] Takeuchi M Yamamoto T. Apoptosis induced by NAD depletion is inhibited by KN-93 in a CaMKII-independent manner. Exp Cell Res. (2015) 335:62–7. 10.1016/j.yexcr.2015.05.019 26024774

[63] Thakur BK Dittrich T Chandra P Becker A Kuehnau W Klusmann JH. Involvement of p53 in the cytotoxic activity of the NAMPT inhibitor FK866 in myeloid leukemic cells. Int J Cancer. (2013) 132:766–74. 10.1002/ijc.27726 22815158 PMC3562481

[64] Wosikowski K Mattern K Schemainda I Hasmann M Rattel B Loser R. WK175, a novel antitumor agent, decreases the intracellular nicotinamide adenine dinucleotide concentration, and induces the apoptotic cascade in human leukemia cells. Cancer Res. (2002) 62:1057–62. 11861382

[65] Cambronne XA Stewart ML Kim D Jones-Brunette AM Morgan RK Farrens DL. Biosensor reveals multiple sources for mitochondrial NAD(+). Science. (2016) 352:1474–7. 10.1126/science.aad5168 27313049 PMC6530784

[66] Yamamoto M Hikosaka K Mahmood A Tobe K Shojaku H Inohara H. Nmnat3 is dispensable in mitochondrial NAD level maintenance *in vivo*. PLoS ONE. (2016) 11:e0147037. 10.1371/journal.pone.0147037 26756334 PMC4710499

[67] Tateishi K Iafrate AJ Ho Q Curry WT Batchelor TT Flaherty KT. Myc-driven glycolysis is a therapeutic target in glioblastoma. Clin Cancer Res. (2016) 22:4452–65. 10.1158/1078-0432.CCR-15-2274 27076630 PMC5010492

[68] Han X Tai H Wang X Wang Z Zhou J Wei X. AMPK activation protects cells from oxidative stress-induced senescence via autophagic flux restoration and intracellular NAD(+) elevation. Aging Cell. (2016) 15:416–27. 10.1111/acel.12446 26890602 PMC4854918

[69] Stein LR Imai S. Specific ablation of Nampt in adult neural stem cells recapitulates their functional defects during aging. EMBO J. (2014) 33:1321–40. 10.1002/embj.201386917 24811750 PMC4194122

[70] Yoshino J Baur JA Imai SI. NAD(+) intermediates: the biology and therapeutic potential of NMN and NR. Cell Metab. (2018) 27:513–28. 10.1016/j.cmet.2017.11.002 29249689 PMC5842119

[71] Fang EF Kassahun H Croteau DL Scheibye-Knudsen M Marosi K Lu H. NAD(+) replenishment improves lifespan and healthspan in ataxia telangiectasia models via mitophagy and DNA repair. Cell Metab. (2016) 24:566–81. 10.1016/j.cmet.2016.09.004 27732836 PMC5777858

[72] Gujar AD Le S Mao DD Dadey DY Turski A Sasaki Y. An NAD+-dependent transcriptional program governs self-renewal and radiation resistance in glioblastoma. Proc Natl Acad Sci USA. (2016) 113:E8247–56. 10.1073/pnas.1610921114 27930300 PMC5187672

[73] Fang EF Lautrup S Hou Y Demarest TG Croteau DL Mattson MP. NAD(+) in aging: molecular mechanisms and translational implications. Trends Mol Med. (2017) 23:899–916. 10.1016/j.molmed.2017.08.001 28899755 PMC7494058

[74] Qu Y Zhang J Wu S Li B Liu S Cheng J. SIRT1 promotes proliferation and inhibits apoptosis of human malignant glioma cell lines. Neurosci Lett. (2012) 525:168–72. 10.1016/j.neulet.2012.07.025 22867969

[75] Sayd S Thirant C El-Habr EA Lipecka J Dubois LG Bogeas A Sirtuin-2 activity is required for glioma stem cell proliferation arrest but not necrosis induced by resveratrol. Stem Cell Rev. (2014) 10:103–13. 10.1007/s12015-013-9465-0 23955573

[76] Funato K Hayashi T Echizen K Negishi L Shimizu N Koyama-Nasu R. SIRT2-mediated inactivation of p73 is required for glioblastoma tumorigenicity. EMBO Rep. (2018) 19:e45587. 10.15252/embr.201745587 30213795 PMC6216266

[77] Li Y Dai D Lu Q Fei M Li M Wu X. Sirt2 suppresses glioma cell growth through targeting NF-kappaB-miR-21 axis. Biochem Biophys Res Commun. (2013) 441:661–7. 10.1016/j.bbrc.2013.10.077 24161395

[78] Chen X Li D Gao Y Cao Y Hao B. Histone deacetylase SIRT6 inhibits glioma cell growth through down-regulating NOTCH3 expression. Acta Biochim Biophys Sin. (2018) 50:417–24. 10.1093/abbs/gmy019 29659670

[79] Feng J Yan PF Zhao HY Zhang FC Zhao WH Feng M. SIRT6 suppresses glioma cell growth via induction of apoptosis, inhibition of oxidative stress and suppression of JAK2/STAT3 signaling pathway activation. Oncol Rep. (2016) 35:1395–402. 10.3892/or.2015.4477 26648570

[80] Chang M Qiao L Li B Wang J Zhang G Shi W. Suppression of SIRT6 by miR-33a facilitates tumor growth of glioma through apoptosis and oxidative stress resistance. Oncol Rep. (2017) 38:1251–8. 10.3892/or.2017.5780 28677777

[81] Tentori L Ricci-Vitiani L Muzi A Ciccarone F Pelacchi F Calabrese R. Pharmacological inhibition of poly(ADP-ribose) polymerase-1 modulates resistance of human glioblastoma stem cells to temozolomide. BMC Cancer. (2014) 14:151. 10.1186/1471-2407-14-151 24593254 PMC3975727

[82] Morgan MA Canman CE. Replication stress: an achilles' heel of glioma cancer stem-like cells. Cancer Res. (2018) 78:6713–6. 10.1158/0008-5472.CAN-18-2439 30498082 PMC6295240

[83] Lu Y Kwintkiewicz J Liu Y Tech K Frady LN Su YT. Chemosensitivity of IDH1-mutated gliomas due to an impairment in PARP1-mediated DNA repair. Cancer Res. (2017) 77:1709–18. 10.1158/0008-5472.CAN-16-2773 28202508 PMC5380481

[84] Murnyak B Kouhsari MC Hershkovitch R Kalman B Marko-Varga G Klekner A. PARP1 expression and its correlation with survival is tumour molecular subtype dependent in glioblastoma. Oncotarget. (2017) 8:46348–62. 10.18632/oncotarget.18013 28654422 PMC5542272

[85] Forment JV O'Connor MJ. Targeting the replication stress response in cancer. Pharmacol Ther. (2018) 188:155–67. 10.1016/j.pharmthera.2018.03.005 29580942

[86] Lecona E Fernandez-Capetillo O. Targeting ATR in cancer. Nat Rev Cancer. (2018) 18:586–95. 10.1038/s41568-018-0034-3 29899559

[87] Woroniecka K Chongsathidkiet P Rhodin K Kemeny H Dechant C Farber SH. T-cell exhaustion signatures vary with tumor type and are severe in glioblastoma. Clin Cancer Res. (2018) 24:4175–86. 10.1158/1078-0432.CCR-17-1846 29437767 PMC6081269

[88] Bryant HE Petermann E Schultz N Jemth AS Loseva O Issaeva N. PARP is activated at stalled forks to mediate Mre11-dependent replication restart and recombination. EMBO J. (2009) 28:2601–15. 10.1038/emboj.2009.206 19629035 PMC2738702

[89] Hanzlikova H Kalasova I Demin AA Pennicott LE Cihlarova Z Caldecott KW. The importance of poly(ADP-Ribose) polymerase as a sensor of unligated okazaki fragments during DNA replication. Mol Cell. (2018) 71:319–31 e313. 10.1016/j.molcel.2018.06.004 29983321 PMC6060609

[90] Maya-Mendoza A Moudry P Merchut-Maya JM Lee M Strauss R Bartek J. High speed of fork progression induces DNA replication stress and genomic instability. Nature. (2018) 559:279–84. 10.1038/s41586-018-0261-5 29950726

[91] Petermann E Orta ML Issaeva N Schultz N Helleday T. Hydroxyurea-stalled replication forks become progressively inactivated and require two different RAD51-mediated pathways for restart and repair. Mol Cell. (2010) 37:492–502. 10.1016/j.molcel.2010.01.021 20188668 PMC2958316

[92] Ying S Hamdy FC Helleday T. Mre11-dependent degradation of stalled DNA replication forks is prevented by BRCA2 and PARP1. Cancer Res. (2012) 72:2814–21. 10.1158/0008-5472.CAN-11-3417 22447567

[93] Lord CJ Ashworth A. PARP inhibitors: synthetic lethality in the clinic. Science. (2017) 355:1152–8. 10.1126/science.aam7344 28302823 PMC6175050

[94] Pommier Y O'Connor MJ de Bono J. Laying a trap to kill cancer cells: PARP inhibitors and their mechanisms of action. Sci Transl Med. (2016) 8:362ps317. 10.1126/scitranslmed.aaf9246 27797957

[95] Almiron Bonnin DA Havrda MC Lee MC Liu H Zhang Z Nguyen LN. Secretion-mediated STAT3 activation promotes self-renewal of glioma stem-like cells during hypoxia. Oncogene. (2018) 37:1107–18. 10.1038/onc.2017.404 29155422 PMC5851110

[96] Lucena-Cacace A Otero-Albiol D Jimenez-Garcia MP Peinado-Serrano J Carnero A. NAMPT overexpression induces cancer stemness and defines a novel tumor signature for glioma prognosis. Oncotarget. (2017) 8:99514–30. 10.18632/oncotarget.20577 29245920 PMC5725111

[97] Lucena-Cacace A Otero-Albiol D Jimenez-Garcia MP Munoz-Galvan S Carnero A. NAMPT is a potent oncogene in colon cancer progression that modulates cancer stem cell properties and resistance to therapy through Sirt1 and PARP. Clin Cancer Res. (2018) 24:1202–15. 10.1158/1078-0432.CCR-17-2575 29203587

[98] Coffey RJ Lunsford LD Taylor FH. Survival after stereotactic biopsy of malignant gliomas. Neurosurgery. (1988) 22:465–73. 10.1227/00006123-198803000-00003 2452376

[99] Aldape K Zadeh G Mansouri S Reifenberger G von Deimling A. Glioblastoma: pathology, molecular mechanisms and markers. Acta Neuropathol. (2015) 129:829–48. 10.1007/s00401-015-1432-1 25943888

[100] Stupp R Mason WP van den Bent MJ Weller M Fisher B Taphoorn MJ. Radiotherapy plus concomitant and adjuvant temozolomide for glioblastoma. N Engl J Med. (2005) 352:987–96. 10.1056/NEJMoa043330 15758009

[101] Zorzan M Giordan E Redaelli M Caretta A Mucignat-Caretta C. Molecular targets in glioblastoma. Future Oncol. (2015) 11:1407–20. 10.2217/fon.15.22 25952786

[102] Nishikawa R Standard therapy for glioblastoma–a review of where we are. Neurol Med Chir. (2010) 50:713–9. 10.2176/nmc.50.713 20885105

[103] Ferrara N Hillan KJ Novotny W. Bevacizumab (Avastin), a humanized anti-VEGF monoclonal antibody for cancer therapy. Biochem Biophys Res Commun. (2005) 333:328–35. 10.1016/j.bbrc.2005.05.132 15961063

[104] Lau D Magill ST Aghi MK. Molecularly targeted therapies for recurrent glioblastoma: current and future targets. Neurosurg Focus. (2014) 37:E15. 10.3171/2014.9.FOCUS14519 25434384 PMC5058366

[105] Galanis E Jaeckle KA Maurer MJ Reid JM Ames MM Hardwick JS. Phase II trial of vorinostat in recurrent glioblastoma multiforme: a north central cancer treatment group study. J Clin Oncol. (2009) 27:2052–8. 10.1200/JCO.2008.19.0694 19307505 PMC2669764

[106] Reardon DA Desjardins A Peters KB Gururangan S Sampson JH McLendon RE. Phase II study of carboplatin, irinotecan, and bevacizumab for bevacizumab naive, recurrent glioblastoma. J Neurooncol. (2012) 107:155–64. 10.1007/s11060-011-0722-2 21986722 PMC3616617

[107] Shabason JE Tofilon PJ Camphausen K. Grand rounds at the National Institutes of Health: HDAC inhibitors as radiation modifiers, from bench to clinic. J Cell Mol Med. (2011) 15:2735–44. 10.1111/j.1582-4934.2011.01296.x 21362133 PMC3112261

[108] Sasaki Y Hackett AR Kim S Strickland A Milbrandt J. Dysregulation of NAD(+) Metabolism induces a schwann cell dedifferentiation program. J Neurosci. (2018) 38:6546–62. 10.1523/JNEUROSCI.3304-17.2018 29921717 PMC6052240

[109] Darmanis S Sloan SA Croote D Mignardi M Chernikova S Samghababi P. Single-cell RNA-Seq analysis of infiltrating neoplastic cells at the migrating front of human glioblastoma. Cell Rep. (2017) 21:1399–410. 10.1016/j.celrep.2017.10.030 29091775 PMC5810554

